# The Clinical Practice Guideline on diagnosis and treatment of hyponatraemia: a response from Otsuka Pharmaceutical Europe Ltd

**DOI:** 10.1530/EJE-14-0392

**Published:** 2014-07

**Authors:** Marco Avila

**Affiliations:** Otsuka Pharmaceutical Europe Ltd Gallions, Wexham Springs, Framewood Road, Wexham, SL3 6PJUK

The publication of the guideline by the European Renal Best Practice (ERBP) group has helped stimulate a debate on this important cause of morbidity and mortality (1, 2), and this joins an ever-growing body of international and national guidelines on hyponatraemia. This includes the international expert panel recommendations by Verbalis *et al*. (3), and various national recommendations from Spain (4), Sweden (5) and the UK (6). The development of hyponatraemia guidelines in Italy and Norway is also ongoing.

Therefore, it seems to be unusual that tolvaptan, the only licensed vasopressin receptor antagonist (vaptan) for hyponatraemia secondary to the syndrome of inappropriate secretion of antidiuretic hormone (SIADH) (1, 2), has been excluded from the European ‘Clinical Practice Guideline on the diagnostic approach and treatment of hyponatraemia’ (1, 2). By contrast, most of the recommended treatments are unlicensed for treating the conditions that they are recommended for and do not have the benefit–risk assessment that is signified by licence approval following the regulatory assessment by the European Medicines Agency (EMA) and the Food and Drug Administration (FDA) (7, 8).

Notably, some of the recommendations of the ERBP guideline are in contrast to those of the other publications mentioned above, particularly in patients with SIADH (1, 2, 3, 4, 5, 6).

Unusually, the authors have chosen to analyse four vaptans (tolvaptan, conivaptan, lixivaptan and satavaptan) within the same meta-analysis (9, 10) when only tolvaptan is licensed and approved for use in Europe (1, 2). Moreover, this analysis is based on a heterogeneous population that includes hypervolaemic patients, whereas in Europe tolvaptan is authorised for use only in patients with euvolaemic hyponatraemia secondary to SIADH (1, 2, 9, 10).

Although there is a paucity of evidence to support evidence-based recommendations for most available therapies in hyponatraemia (3), the efficacy and safety profile of tolvaptan have been proven in randomised controlled trials (11). Furthermore, since 31 March 2013, pooled exposure data have been made available for the ongoing evaluation of safety from 82 trials, including 6794 subjects worldwide who were exposed to oral doses of tolvaptan, with 425 subjects in trials for hyponatraemia (12). Therefore, it is surprising that the European guideline does not recommend a place for tolvaptan within its licensed indication (1, 2). Based on their review of a meta-analysis consisting predominantly of unlicensed vaptans, the authors concluded that the benefit–risk profile for vaptans seems to be negative. Vaptans, similar to all other treatments for hyponatraemia, can occasionally cause overly rapid correction of hyponatraemia (11). A single case of osmotic demyelination syndrome (13), with a very rare incidence of less than one in 10 000 patients, has been reported with tolvaptan monotherapy to date. The summary of product characteristics for tolvaptan emphasises the importance of close monitoring of serum sodium concentration and titrating the dose to achieve a desired serum sodium concentration in all patients (7).

The authors also expressed concern with the hepatotoxicity associated with the use of particularly high doses of tolvaptan in autosomal dominant polycystic kidney disease (ADPKD) (1, 2). However, there have been no liver safety signals to date in any other population treated with tolvaptan (hyponatraemia, heart failure or cirrhosis) (14). The long-term safety profile of tolvaptan has been confirmed in the SALTWATER study, with a mean duration of treatment of 1.9 years (15).

The vaptan meta-analysis also suggested a signal indicating a possibly increased risk of death for hypervolaemic patients treated with vaptans compared with placebo (1, 2). Moreover, it is important to note that in Europe tolvaptan is not approved in this patient population (1, 2) and there was no increase in mortality when the tolvaptan subgroup was analysed (9, 10).

With the rigour afforded to the analyses of the risk–benefit profile for vaptans, it is surprising that the same in-depth analyses do not seem to have been conducted for any treatments the ERBP guideline recommends for SIADH. The authors acknowledge that there are no systematic reviews or randomised controlled trials evaluating the benefits and harms of fluid restriction, urea or loop diuretics (1, 2).

Yet the authors' recommendation for first-line treatment with fluid restriction fails to acknowledge that it may be inappropriate for many patient populations (e.g. in patients with cancer where it may delay chemotherapy (16, 17), and it is likely to fail in some patients (e.g. those with high urine osmolality) (3).

The guideline recommends second-line treatment with urea or the combination of loop diuretics with oral sodium chloride (NaCl), despite extremely limited efficacy data (1, 2). The authors failed to consider safety data for the use of urea in the studies they quote, for example, one study has shown that urea is associated with a 37% rate of overly rapid correction in patients with severe hyponatraemia (18). In addition, of the four studies mentioned supporting the use of loop diuretics with oral NaCl (19, 20, 21, 22), only one study actually used oral NaCl (*n*=9), with the remaining studies using NaCl intravenously (19). There is no discussion on the rationale for the inclusion of this combination or any consideration of safety data (1, 2), for example, overly rapid correction was observed in 56–80% of patients in two case series (20, 22), and hypokalaemia developed in 58% of patients in another study (19).

Tolvaptan has a proven benefit–risk profile, which is supported by randomised controlled trials and over 4 years of real-world use, and we urge the authors to take a more consistent and measured approach towards evidence-based recommendations.

## Reply to this letter

A response to this letter (23) is being published in this issue.

## Co-publication

This letter will be co-published in the *European Journal of Endocrinology* and *Nephrology Dialysis Transplantation*.
